# My Experience with Median Neurectomy1Read before the Veterinary Medical Association of New Jersey at a regular meeting held in Newark, N. J., May 10, 1900.

**Published:** 1900-06

**Authors:** J. Payne Lowe

**Affiliations:** Passaic, N. J.


THE JOURNAL

OF

COMPARATIVE MEDICINE AND

VETERINARY ARCHIVES.

VOL. XXI. *JUNE, 1900.* No. 6.

### MY EXPERIENCE WITH MEDIAN NEURECTOMY.[1]

By J. Payne Lowe, D.V.S.,
PASSAIC, N. J.

In bringing before you the subject of median neurectomy it is my intention mainly to give you my experience with the operation; to be modest, to cite a number of cases which I have operated upon during the past year or two, giving you accurately and impartially the pathological conditions for which the operation was performed in each case, the immediate result, and the subsequent history to date; in doing this I hope to be able to show that median neurectomy is an operation that is practicable and safe, and that the practitioner should perform it without hesitancy wherever it is indicated.

Familiarize or rather refresh your mind with the surgical anatomy of the region, which you can get from any of the standard authorities on veterinary anatomy. The anatomical essentials, however, can probably be more readily obtained by referring directly to that most excellent treatise on this operation by Professor C. Pellerin, and translated into English by our own Liautard.

Where the operating-table cannot be used the animal is cast with the hobbles on the side which is to be operated upon; the upper forelimb is taken from the hobble, and by means of a side line secured to the corresponding hind one. The lower forelimb (the one to be operated upon) is also taken from its hobble, and the bar hobble applied to it and the shin of the hind limb. This limb is also held firmly in extension by an assistant with a strap or rope. Having thus well secured your animal, have sponges, antiseptics, solutions, etc., and the necessary instruments ready, which are: two bistouries, one convex and one straight, a grooved directory,

[1] Read before the Veterinary Medical Association of New Jersey at a regular meeting held in Newark, N. J., May 10, 1900.

curved scissors, bull-dog forceps, plain dissecting forceps, a couple of blunt retractors, and a blunt tenaculum.

The operation is performed upon the inner side and upper portion of the forearm; the point for the incision is immediately under the round eminence formed superiorly by humero-radial articulation, and inferiorly by bicipital tuberosity and the rough impressions back of it. Placing the finger just inferior to the above eminence, feel for the posterior border of the radius, and make your incision just a little posterior to that border, say a quarter of an inch. By observing this point you will generally be right over the nerve and posterior to the vein. At this point make your incision through the skin, about one and a half inch long, then cut through and parallel with the fibres of the sterno-aponeuroticus muscle; then go through the cellular tissue, when you will come down upon the antibrachial aponeurosis, which is attached to the posterior border of the radius, and is dense and inelastic. Now carefully take hold of this aponeurosis with your bull-dog forceps, raising it slightly, and carefully puncture it sufficiently to allow the introduction of the grooved directory, using this as a guard against injuring the vein. By cutting from within outward, divide this aponeurosis to the extent of your outer incision. Have an assistant hold the edges of the wound apart with a pair of blunt retractors. We now see the internal flexor of the metacarpus. In front of this muscle we should see the nerve which is recognized by its whitish color, flattened form and sensitiveness. You now also see the large vein in front of the nerve, which is recognized by its dark color. At this location the vein usually lies in front of the nerve. The artery is generally concealed by the vein and nerve; but experience and a careful study of the anatomy will show that these relations are not always constant, so if you do not see the nerve have patience and look further.

Having found and isolated the nerve, pass a blunt tenaculum under it and raise it slightly, dividing it on a level with the superior part of your incision. Take hold of the distal end and resect an inch of the nerve and remove it. Wash your wound out antiseptically, and your operation is completed.

If the operation has been neatly performed the wound will be healed in about two weeks. If the season permits irrigate the limb daily with cold water, and follow out the general principles of antiseptics.

Case I.—The subject was an aged mare, weighing about one thousand pounds, suffering with chronic periostosis of the fetlock.

The lameness was very marked. I operated upon her on April 1, 1899. Immediately after the operation an apparent improvement was noticed, which was probably due to shock and being confined in the hobbles. On the second day she was very stiff and sore in walking, which was due to the soreness and swelling in the operated region. This animal was given six weeks' rest, but the results were negative. No improvement whatever, as the exostosis affected the mobility of the joint. It can be stated that the lameness was mechanical, and therefore could not be overcome.

CASE II.—Small mare used for road purposes. Had been lame in the foot for a period of three months, during which time she rested and received a liberal amount of palliative treatment, and had three blisters applied without any or very little improvement. Cocaine injected over the internal plantar nerve on several occasions caused the mare to go temporarily sound. My diagnosis was navicular disease in its incipiency. I operated on her on April 10, 1899. In two weeks the wound was healed, and in three weeks the mare was driven and went sound, and continues to go sound at this writing. Previous to her lameness this mare had good knee action, and it is interesting to know that after the operation she had all her former action, but she always pointed the foot as she did before the operation. I am of the opinion that the operation diminished the pain sufficiently to remove the lameness, but as sensation was still partial in the foot, and in view of the nature of the regions, she still pointed. I have been looking for this mare to go lame, but she does not.

CASE III.—Mare, twelve years old, weighing about thirteen hundred pounds; had been lame for about a year; was too lame to be of any service, even for slow work, and inspection and manipulation of the limb revealed the following: marked induration of the flexor tendons and contraction of the same, with consequent knuckling; an exostosis was detected on the inside of the coronet; the heel of the foot was high, and in standing the toe alone was placed on the ground; the heel did not quite touch the ground in walking. I looked upon the exostosis as the primary cause of the lameness, and the thickening and contraction of the tendons and the high heel as the result.

On April 15, 1899, I operated upon this mare, and immediately after the operation she stood down square and put weight on the limb in standing; in fact, she had a tendency to rest the other limb. This mare had a month's rest, and since has been serviceably and practically free from lameness. Of course, considering the lesions that had existed we could not and did not get that freedom of movement which you get in a normal limb.

CASE IV.—Mule; chronic tendonitis; very lame, even in walking; limb flexed in standing, and did not get the heel down right in walking; subjected to the operation on May 14, 1899. The usual rest was given; the immediate effect was not so pronounced, but after a month the mule was put to work, going nearly all right, and gradually improved, and now, almost a year afterward, is going serviceably sound, and is again a useful working animal.

CASE V.—Heavy draught-horse, fourteen years old, and weighing about sixteen hundred pounds. A tendonitis developed, which resisted the usual forms of treatment. Rest was given and two blisters applied, but the trouble progressed, contraction and thickening of the tendons becoming well marked, with knuckling. The animal was now very lame, and three months had elapsed.

On September 26, 1899, median neurectomy was resorted to, and at once relief was afforded. The animal rested about six weeks and was put to work, doing heavy hauling, and to-day is going all right. The lesions are still apparent to the eye, but an apparently doomed horse was restored to usefulness.

CASE VI.—Aged gray mare, very lame in walking; periostosis of the fetlock was present. She also had side bones on both forefeet and on one hind foot. I did not connect the lameness with the side bones, but attributed it to the periostosis of the fetlock. I operated on January 15, 1900. Immediately after the operation a marked improvement, but still slightly lame. Was rested for four weeks and seemed to be doing well. Was put to work at heavy pulling and the lameness returned with all its severity. This was a negative result. At this time the animal was disposed of, and had passed out of my observation.

CASE VII.—Mule, lame for a year or more, lameness gradually becoming aggravated. On February 12, 1900, at the time of the operation, was very lame and not much weight put on the limb when standing. Immediately after the operation a marked improvement was noted in walking and trotting, though the action of the lower part of the limb was limited. The last time I saw this animal he was going well and working every day. I have operated on several other cases quite recently, but at this time will not report on them, as I have not had an opportunity of seeing the results. You will therefore see that in two cases of chronic periostosis of fetlock the result in both cases was negative, but some anchylosis was present, hence a mechanical lameness remained.

One case of navicular disease; result successful. I do not wish it to be interpreted that I prefer this operation for navicular arthritis, but the case was experimental and I give the results.

Four cases of chronic tendonitis; all successful. Some advocates of the operation claim that it is indicated in many of the chronic conditions from the knee down. Exostosis on the knee, canon, fetlock, and foot, providing no anchylosis is present, and the lesions are on the inner side of the limb. About many of these I cannot express an opinion, as I have not operated upon this class of cases; but there is a class of chronic cases often seen in heavy horses (chronic tendonitis) in which condition the animal is practically useless, where firing and blistering are often ineffective, and I do believe that in this class of cases that if you perform median neurectomy you will be rewarded by very gratifying results.

In concluding I would say that you must use good judgment in the selection of your cases. Do not select cases where it is not indicated, and then condemn the operation because the results are negative. And, again, on the other hand, do not operate on cases where it is unnecessary and where other forms of treatment would give relief.

